# Hepatic Duct Foreign Body Mimicking Cholangiocarcinoma: Case Analysis, Diagnostic Challenges, and Literature Review

**DOI:** 10.7759/cureus.98361

**Published:** 2025-12-03

**Authors:** Ioannis Braimakis, Foteini Leventaki, Nikolaos Papadopoulos, Panagiotis Tsibouris, Periklis Apostolopoulos

**Affiliations:** 1 Translational Gastroenterology and Liver Unit, John Radcliffe Hospital, Oxford University Hospitals, Oxford, GBR; 2 Gastroenterology Department, 417 Army Equity Fund Hospital (NIMTS), Athens, GRC; 3 Second Department of Internal Medicine, 401 General Military Hospital of Athens, Athens, GRC

**Keywords:** cholangiocarcinoma, cholangioscopy, ercp, foreign body, mrcp

## Abstract

Foreign bodies in the biliary tract may provoke an inflammatory reaction, leading to lithogenesis followed by biliary obstruction with or without cholangitis. Such occurrences are typically associated with previous biliary surgery or endoscopic interventions, particularly cholecystectomy or endoscopic retrograde cholangiopancreatography (ERCP). Imaging studies, which usually provide valuable insights into biliary anatomy, may fall short in more difficult cases, making direct visualization of the biliary tree necessary.

We present a case of an impacted bile duct stone in the right hepatic duct formed around a retained fragment of ERCP equipment left behind several years earlier. The patient presented with cholestasis and cholangitis. Magnetic resonance cholangiopancreatography (MRCP) revealed a bile duct stone embedded within a solitary hepatic duct stenosis, findings that were highly suspicious for the presence of cholangiocarcinoma. Thus, cholangioscopy was performed excluding malignancy and identifying the culprit. This procedure allowed the direct visualization of the stricture and acquisition of multiple biopsies. After hepatic duct dilatation and stone retrieval, a foreign body was identified in the stone core. The patient had rapid clinical improvement after stone extraction and remained asymptomatic during follow-up.

In conclusion, foreign bodies should also be part of biliary obstruction differential diagnosis, and no diagnostic modality should be excluded when pursuing a definite diagnosis.

## Introduction

Endoscopic retrograde cholangiopancreatography (ERCP), first introduced in 1968, has long been considered the modality of choice for therapeutic interventions in the biliary tract [1,2]. Among ERCP indications, the most common are the extraction of common bile duct (CBD) and intrahepatic stones, biliary drainage in both benign and malignant strictures, and management of postoperative biliary leaks [2]. Widespread therapeutic implementation of ERCP procedures has unraveled several post-ERCP adverse events [3] as well as technical limitations related to the procedure's inability to directly visualize the biliary tree [4]. To address this pitfall, cholangioscopy has been developed [5]. This technique permits the direct visualization of the bile duct for diagnostic and therapeutic purposes [5]. It allows the management of difficult stones under direct visualization using lithotripsy (electrohydraulic or laser) [6]. Moreover, the use of cholangioscopy has facilitated the diagnosis of indeterminate bile duct strictures, allowing both the visual inspection of stricture characteristics and the acquisition of targeted biopsies [7]. Cholangioscopy-guided biopsies have demonstrated a sensitivity and specificity for malignancy of 69% and 98%, respectively, whereas direct visualization of the bile duct has shown sensitivity and specificity rates of 90% and 87%, respectively [7]. In general, the use of cholangioscopy in indeterminate biliary lesions can increase the adoption of conservative management over surgical intervention [8].

Foreign bodies are rarely encountered in the biliary tract, and relevant data are scarce. Most reported cases have been attributed to the retention of surgical and endoscopy paraphernalia like gauzes and surgical clips, stents, fractured guidewires, and Dormia baskets, but also fish bones, bird feathers, worms, and ingested metal pins [9-16]. Presumably, some of these entered the CBD retrogradely, while others penetrated the walls of the duodenum and CBD. Moreover, some migrated within the biliary tract after laparoscopic cholecystectomy, while others represent retained ERCP equipment [9,10,15,17]. The clinical presentation is usually characterized by biliary pain, fever, and jaundice [10]. Yu et al., reviewing all reported cases of choledocholithiasis caused by foreign bodies, concluded that ERCP is the modality of choice for their removal, outperforming operative or conservative treatment [10].

Herein, we report a case of a patient admitted with cholangitis and an intraluminal filling defect found in the magnetic resonance cholangiopancreatography (MRCP). Subsequent endoscopic evaluation with ERCP and cholangioscopy revealed that the lesion was a bile duct stone formed around a fragment of an instrument retained during an ERCP procedure performed 14 years earlier.

## Case presentation

**Figure 1:**
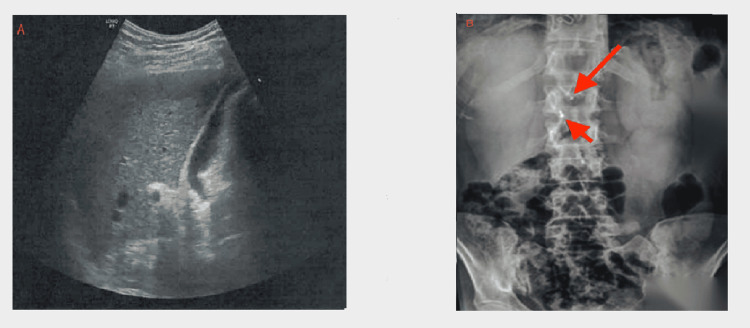
(A) Ultrasound showing normal liver parenchyma without biliary dilatation. (B) Plain radiography showing post-cholecystectomy clips (small arrow) and a linear radiopaque tubular structure (large arrow) in the right upper quadrant

A 58-year-old woman presented to the emergency department with fever up to 38.9°C, persistent epigastric pain, and nausea. The patient was on medical treatment for Hashimoto's thyroiditis and depression. Fourteen years ago, she had had an open cholecystectomy after a successful ERCP, but detailed procedural records were not available. She did not consume any alcohol, and she was a non-smoker. Physical examination revealed upper abdominal tenderness and distention, without any signs of peritonitis. Bowel sounds were audible. Blood work, upon presentation, showed elevated inflammatory markers (C-reactive protein (CRP) 9.3 mg/dL), as well as elevated aminotransferases (aspartate aminotransferase (AST) 508 IU/L and alanine aminotransferase (ALT) 656 IU/L) and cholestatic enzymes (alkaline phosphate (ALP) 125 IU/L and gamma-glutamyl transferase (GGT) 192 IU/L) (Table 1). White blood cell count and total bilirubin were normal (Table 1). Upper abdominal ultrasound was unremarkable, while abdominal plain radiography revealed a tubular, unidentified object in the upper right quadrant (Figure 1A-1B).

**Table 1:** Blood test results Reference ranges are provided for comparison. –: not available; NA: not applicable; ERCP: endoscopic retrograde cholangiopancreatography; WBC: white blood cell; Hb: hemoglobin; PLT: platelet; CRP: C-reactive protein; AST: aspartate aminotransferase; ALT: alanine aminotransferase; ALP: alkaline phosphatase; GGT: gamma-glutamyl transferase; INR: international normalized ratio; PT: prothrombin time; aPTT: activated partial thromboplastin time; HBsAg: hepatitis B surface antigen; anti-HBc: anti-hepatitis B core antibody; anti-HCV: anti-hepatitis C virus antibody; anti-HAV: anti-hepatitis A virus antibody; CMV IgM: cytomegalovirus immunoglobulin M; EBV VCA IgM: Epstein-Barr virus viral capsid antigen immunoglobulin M; HSV 1 IgM: herpes simplex virus type 1 immunoglobulin M; HSV 2 IgM: herpes simplex virus type 2 immunoglobulin M; CA-19-9: carbohydrate antigen 19-9; IgA: immunoglobulin A; IgG: immunoglobulin G; IgM: immunoglobulin M

Parameter	Upon first admission	Before the first ERCP/SpyGlass™	Before the second ERCP/SpyGlass™	Reference range
WBC, K/μL	6.2	3.6	3.5	4-10
Hematocrit (%)	41.3	40.6	43	38-52
Hb, g/dL	14.4	13.7	14.4	13-18
PLT count, K/μL	195	235	271	140-450
CRP, mg/dL	9.3	3.6	3.4	<0.5
Direct bilirubin, mg/dL	0.9	0.8	0.7	<0.5
AST, IU/L	508	89	15	5-40
ALT, IU/L	656	173	22	5-40
ALP, IU/L	125	93	56	40-140
GGT, IU/L	392	364	59	10-50
Amylase, IU/L	36	50	59	25-125
Creatinine, mg/dL	0.8	0.7	0.7	0.72-1.25
Urea, mg/dL	18	22	27	10-55
Total protein, g/dL	7.0	6.7	–	6.4-8.3
Albumin, g/dL	3.8	3.4	–	3.4-5
INR	1.2	1.0	0.99	0.8-1.2
PT, sec	12.2	11.5	11.1	11-14
aPTT, sec	26.4	28	25.4	25-35
HBsAg	–	–	–	0.00-1.00
Anti-HBc	–	–	–	0.00-1.00
Anti-HCV	–	–	–	0.00-1.00
Anti-HAV	–	–	–	0.00-1.00
CMV IgM, IU/mL	5.6	–	–	NA
EBV VCA IgM	6.97	–	–	NA
HSV 1 IgM	2.45	–	–	NA
HSV 2 IgM	–	–	–	NA
IgA, mg/dL	102	–	–	80-450
IgG, mg/dL	847	–	–	750-1560
IgM, mg/dL	119	–	–	45-305
CA-19-9, U/ml	112	-	-	0-37

**Figure 2:**
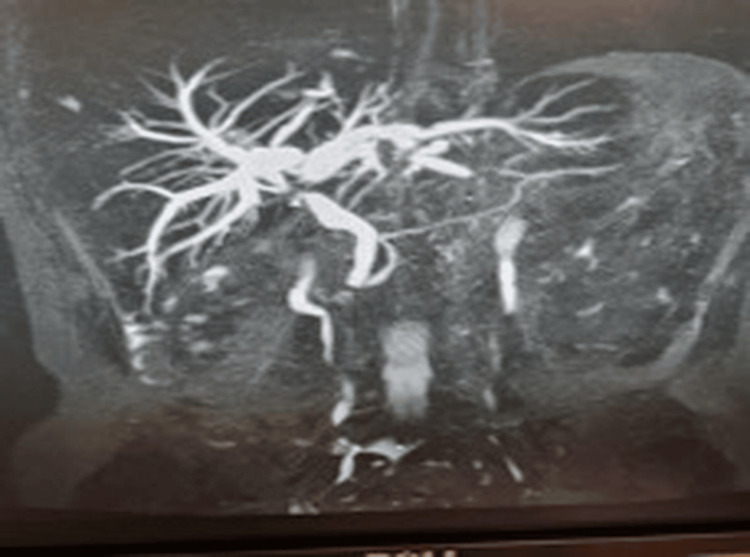
MRCP before the first ERCP/SpyGlass™. Intraluminal filling defect is observed close to the junction of the right and left hepatic ducts MRCP: magnetic resonance cholangiopancreatography; ERCP: endoscopic retrograde cholangiopancreatography

The patient was admitted to the Internal Medicine ward for further investigation. Immunological tests for viral and autoimmune hepatitis were negative, as well as antimitochondrial antibodies (AMA) and immunoelectrophoresis, but serum carbohydrate antigen 19-9 (CA-19-9) was mildly elevated (Table 1). Contrast-enhanced upper abdominal magnetic resonance imaging (MRI) and MRCP demonstrated an intraluminal filling defect near the confluence of the left and right hepatic ducts, accompanied by dilation of the intrahepatic bile ducts. The defect had well-defined margins and low T2 signal intensity. An 18-mm tissue with unclear boundaries that was unevenly enhanced when contrast was administered was found peripherally to the filling defect. In addition, a 14.5-mm-diameter lymph node was found at the porta hepatis. CBD was normal (width of 7.5 mm) (Figure 2). 

**Figure 3:**
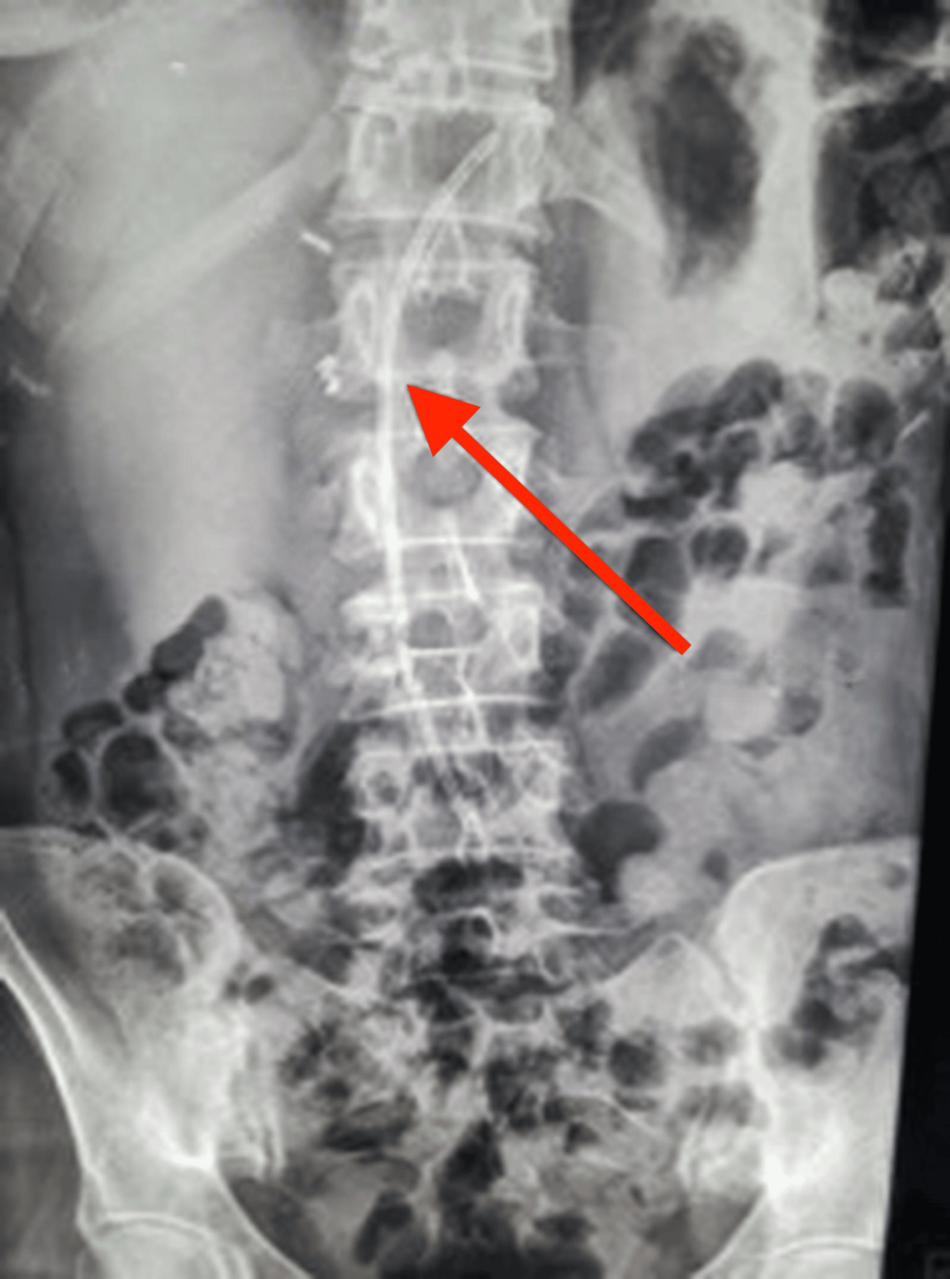
Abdominal plain radiography after the first ERCP/SpyGlass™. A plastic stent (arrow) placed in the first ERCP, post-cholecystectomy clips, and an unidentified tubular radiopaque object are observed ERCP: endoscopic retrograde cholangiopancreatography

According to the Tokyo Guidelines (TG18), the presence of systemic inflammation together with biochemical cholestasis in the absence of organ dysfunction was consistent with Grade I (mild) acute cholangitis [17]. Simultaneously, the MRI/MRCP findings were highly suspicious for cholangiocarcinoma, and plain radiography findings remained inconclusive. Thus, further evaluation with ERCP and cholangioscopy using the SpyGlass™ DS system (Boston Scientific, Marlborough, Massachusetts, United States) was deemed necessary. During the procedure, the stricture appeared in the right hepatic duct, directly above the junction of the left and right hepatic ducts. Direct visualization revealed a short, smooth, concentric narrowing without mucosal irregularity or neovascularization, features suggesting a benign stricture. Biopsies obtained, using a SpyBite™ biopsy forcep (Boston Scientific, Marlborough, Massachusetts, United States), were negative for cholangiocarcinoma. A bile duct stone was identified above the stricture, at the level where fluoroscopy had revealed a thin radiopaque formation. An attempt to remove the intraductal stone with a balloon catheter was unsuccessful, and an 8.5-French (FR) plastic stent (8.5FR×15 cm) was placed to ensure biliary drainage (Figure 3). Following the procedure, the patient was asymptomatic, and her blood test results gradually improved. 

**Figure 4:**
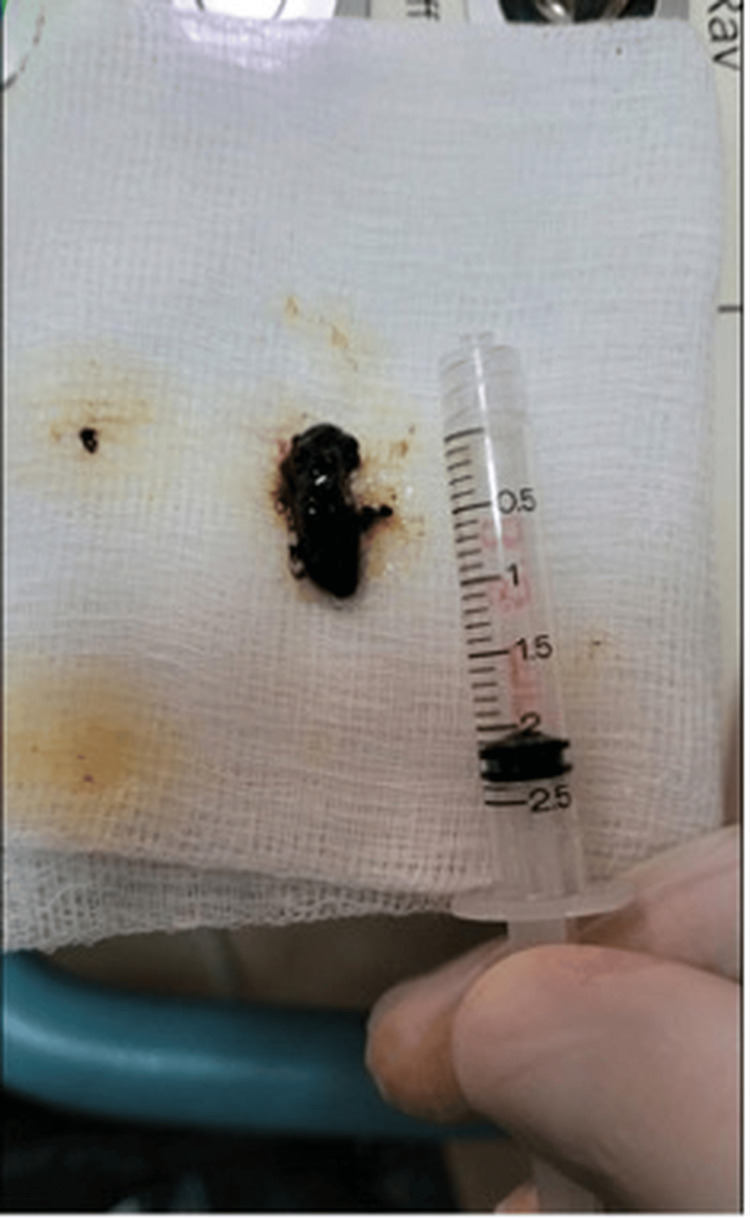
Bile duct stone extracted and retrieved using a retrieval basket at the second ERCP/SpyGlass™ ERCP: endoscopic retrograde cholangiopancreatography

**Figure 5:**
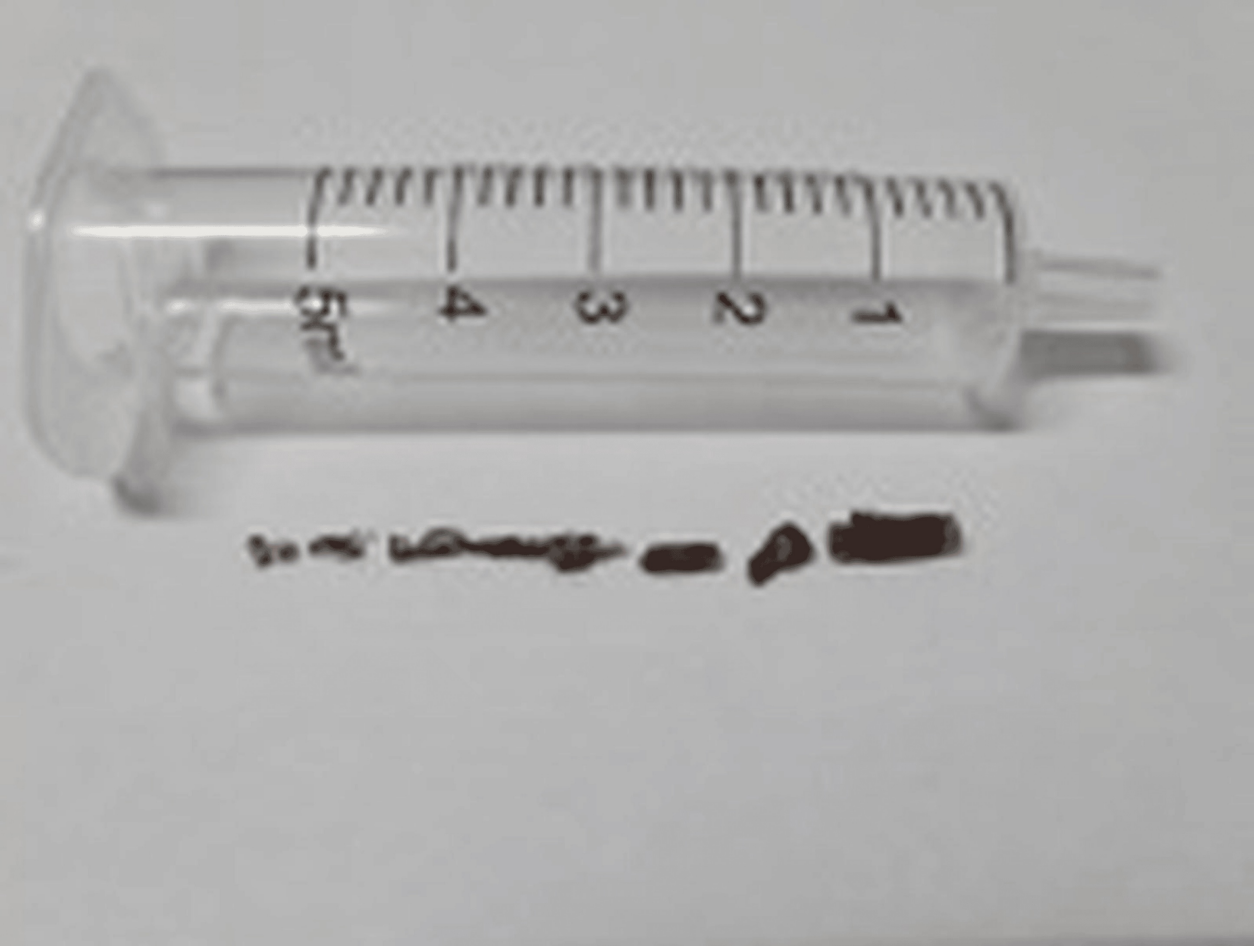
The core fragment showing the tubular shape and rigid material characteristics typical of ERCP catheter components ERCP: endoscopic retrograde cholangiopancreatography

Three months later, a second ERCP attempt was decided because pathology results from the initial biopsies had revealed features of cholangitis without evidence of malignancy. In the meantime, the patient was asymptomatic, and her blood tests were normal (Table 1). During the ERCP, the plastic stent was removed using a snare, followed by repeat cholangioscopy with the SpyGlass™ system. The impacted bile duct stone was again identified in the right hepatic duct. Cannulation of the right hepatic duct with a guidewire was achieved under direct visualization to navigate the wire next to the stone and successfully extract it using a small balloon catheter (8.5 mm). After stone extraction, occlusion cholangiography excluded hepatic duct stenosis, while fluoroscopy ruled out the presence of any tubular radiopaque object. A final overview of the right hepatic duct using SpyGlass™ revealed no stricture and excluded, once more, any hint of malignancy. The bile duct stone partially fragmented during retrieval with the Dormia basket, which allowed a closer inspection of its internal structure and revealed a foreign body resembling a fragment of an ERCP catheter (Figures 4-5). 

As the second ERCP was uneventful, the patient was discharged the following day. At the three-month follow-up, she remained asymptomatic, and repeat MRI/MRCP showed no evidence of intraluminal filling defect, biliary stenosis, or dilation. Also, the previously noted porta hepatis lymph node did not persist on follow-up imaging, supporting a reactive etiology. Similarly, CA-19-9 values returned to normal, consistent with the resolution of inflammation. Three years later, the patient remains under close surveillance without clinical symptoms and without any indication of bile duct stone recurrence.

## Discussion

Obstruction of the biliary tree by a foreign body, followed by lithogenesis at that level, is uncommon and has only rarely been referred to in the literature [9-15]. The most prevalent foreign bodies identified are related to postoperative [9-14] or post-endoscopy complications [9,10]. The vast majority of these present after cholecystectomy or ERCP [10]. Sporadic reports have identified that parasites, fish bones, bird feathers, metal pins, and rubber objects could provide the lithogenic core [10], but more commonly the nucleus for the formation of stones or molds in the biliary tree are shrapnel, metal clips, migrated stents, surgical gauze, and non-absorbable suture material [9-15]. Foreign bodies can also transmigrate into the biliary tract from the duodenum [10]. 

In our case, part of an ERCP catheter served as the lithogenic core. Previously, biliary stents [10,16] and a Dormia basket [9,10,16] have been implicated in stone formation within the biliary tree. The long-time interval between the patient's prior ERCP and the onset of biliary symptoms makes our case particularly challenging. Previous cases of post-ERCP retained foreign objects consisted mainly of impacted baskets [9,10], stents [10], and broken guidewires [10], with symptoms typically appearing after shorter intervals. Nevertheless, as Kitamura et al. [18] have pointed out, symptoms can develop anytime between 11 days and 20 years after the initial foreign body entrapment. An inflammatory reaction with fever and jaundice represents the most frequent clinical presentation in the majority of cases [16], but exceptions such as eosinophilia [14] have also been described. In our case, the inflammatory response was significant, but jaundice was absent as the lesion was located in the right hepatic duct. The differential diagnosis of foreign bodies in the biliary tract is generally challenging. While imaging modalities such as contrast-enhanced MRI and MRCP provide critical insights into anatomical abnormalities and aid in forming initial hypotheses, they may not always provide a comprehensive understanding of the underlying pathology, as they lack real-time visualization capability of dynamic processes. This highlights the complementary role of endoscopy, which enables direct visualization, targeted interventions, and confirmatory diagnoses [14]. Regardless of the type of foreign body encountered, ERCP has been identified as the optimal method for extraction from the biliary tree [10]. The addition of cholangioscopy further maximizes both the diagnostic and therapeutic yield of ERCP [19,20]. 

Despite its diagnostic and therapeutic potential, ERCP is not always uneventful. Complications such as bile leak, post-ERCP pancreatitis, recurrence of biliary tract stones, and even death have been described [3]. In our case, the inflammatory reaction and stenosis distal to the site of the foreign body within the biliary tree were misinterpreted as possible malignancies on MRCP, particularly since they were accompanied by lymphadenopathy at the porta hepatis. Although a plain abdominal X-ray could have provided a clue, as in the majority of reported cases [9-16], it was overlooked in favor of the more detailed findings of abdominal MRI/MRCP. Wen et al. [15] reported a case in which a suture needle led to an inflammatory reaction within the intrahepatic biliary tree resembling a liver neoplasm. As in our case, ERCP provided the definitive diagnosis. A negative cholangiographic appearance, negative pathological results, and, most importantly, an uneventful follow-up with the disappearance of MRI findings after successful ERCP treatment permitted the safe exclusion of neoplasia. 

As indicated by our case, the differentiation between a malignant stricture of the biliary tree and a benign stricture of any etiology, including the presence of a foreign body, is not always evident. MRI and MRCP are valuable tools in this differentiation, with malignant strictures typically characterized by irregular margins and asymmetric narrowing, whereas benign strictures are usually short and symmetric and have regular margins [20]. However, imaging alone may be insufficient, and further histopathological evaluation is essential to establish or exclude cholangiocarcinoma, with tissue samples obtained either via endoscopic ultrasound-guided fine-needle aspiration (EUS-FNA), ERCP brush cytology [20], or cholangioscopy-guided biopsy [7]. 

The extraction of foreign objects from the biliary tract is often challenging. Di Mitri et al. [19], for instance, needed mechanical lithotripsy to remove a fractured distal end of a Dormia basket. Nevertheless, the use of the SpyGlass™ system is sufficient in the majority of cases [14]. In addition, balloon sweeping of the CBD can be performed using a Fogarty balloon catheter, while SpyBite™ forceps may be employed to retrieve foreign objects when other methods fail [19].

## Conclusions

Foreign bodies in the bile duct are a rare but important cause of biliary obstruction and/or cholangitis and should be considered in patients presenting with compatible symptoms, even many years after procedures such as cholecystectomy or ERCP. Notably, accurate evaluation and optimal management are best achieved through the direct visualization of the biliary tract using cholangioscopy, which not only facilitates the extraction of foreign bodies but also allows for the reliable exclusion of other coexisting lesions.
